# The Nutritional Content of Prey Affects the Foraging of a Generalist Arthropod Predator

**DOI:** 10.1371/journal.pone.0049223

**Published:** 2012-11-08

**Authors:** Jason M. Schmidt, Peter Sebastian, Shawn M. Wilder, Ann L. Rypstra

**Affiliations:** 1 Department of Zoology, Miami University, Oxford, Ohio, United States of America; 2 Department of Zoology, Miami University, Hamilton, Ohio, United States of America; Hungarian Academy of Sciences, Hungary

## Abstract

While foraging theory predicts that predatory responses should be determined by the energy content and size of prey, it is becoming increasingly clear that carnivores regulate their intake of specific nutrients. We tested the hypothesis that prey nutrient composition and predator nutritional history affects foraging intensity, consumption, and prey selection by the wolf spider, *Pardosa milvina*. By altering the rearing environment for fruit flies, *Drosophila melanogaster*, we produced high quality flies containing more nitrogen and protein and less lipid than low quality fruit flies. In one experiment, we quantified the proportion of flies taken and consumption across a range of densities of either high or low quality flies and, in a second experiment, we determined the prey capture and consumption of spiders that had been maintained on contrasting diets prior to testing. In both cases, the proportion of prey captured declined with increasing prey density, which characterizes the Type II functional response that is typical of wolf spiders. Spiders with similar nutritional histories killed similar numbers of each prey type but consumed more of the low quality prey. Spiders provided high quality prey in the weeks prior to testing killed more prey than those on the low quality diet but there was no effect of prior diet on consumption. In the third experiment, spiders were maintained on contrasting diets for three weeks and then allowed to select from a mixture of high and low quality prey. Interestingly, feeding history affected prey preferences: spiders that had been on a low quality diet showed no preference but those on the high quality diet selected high quality flies from the mixture. Our results suggest that, even when prey size and species identity are controlled, the nutritional experience of the predator as well as the specific content of the prey shapes predator-prey interactions.

## Introduction

Recently, food web linkages have been viewed through a nutritional lens where quality, as defined as the relative concentrations of critical elements or nutrients in food, provides the framework for understanding animal interactions [1]–[6]. While the energy in prey has long been hypothesized as the primary prey currency influencing predator foraging behavior (reviewed by [7]), it is now clear that the relative amounts of various nutrients in the diet affect the activities of carnivores as well as their performance through subsequent impacts on growth, development, distribution, and survivorship [8]–[13]. Presumably as a result, some arthropod predators regulate food intake in subtle ways in order to maximize nourishment and avoid the negative costs associated with the over consumption of imbalanced prey [10], [14], [15]. Since generalist predators can have large impacts on the structure and function of communities and ecosystems [16], [17], further dissection of the role of carnivore diet and prey nutritional quality on foraging intensity and prey selection is indispensible to any attempt to characterize the relationships between predators and their prey.

Spiders are common generalist predators and, as such, comprise an important component of most terrestrial food webs [16], [18]–[20]. In addition, evidence is accumulating that spider foraging behavior is affected by their dietary history as well as the nutritional value of prospective prey [21]. Indeed, one of the earliest studies in nutritional ecology demonstrated that wolf spiders select prey in order to optimize their intake of essential amino acids [22]. More recent studies confirm that wolf spiders are sensitive to their nutritional needs when deciding whether to capture a particular prey item and, once it is captured, they also modulate the amount they ingest as a way to match their intake to metabolic needs [10], [14]. These unexpected dietary adjustments cause complex interactions among hunger, prey density and predator experience to emerge when wolf spiders are presented nutritionally distinct prey repeatedly over a period of several days [23]. Clearly nutrition colors the activity of wolf spiders and, if we are to understand their role in food webs, we must determine if and how their dietary history as well as the value of the prey available to them at any given moment affect capture frequency, consumption and prey selection.

Here we examine if and how the predatory interactions between the wolf spider, *Pardosa milvina*, and its prey are affected by nutritional differences. While previous studies of *P. milvina* have revealed some effects of hunger, prey size and predation risk on foraging [24]–[26], Schmidt and colleagues [27] were unable to explain prey selection patterns in field studies even when they made every effort to control for those other factors. Specifically, molecular gut analyses revealed that consumption of flies (Diptera) is lower than predicted when flies are abundant and higher than expected when they are scarce [27]. The explanation for this result that emerges from nutritional ecology is that the capture, consumption and selection of prey are influenced by some interplay between the dietary history of the predator and nutritional content of the prey. We delved into this interaction by characterizing the relationship between predation intensity and the availability of prey for *P. milvina* (e.g., the functional response of Holling [28]) and documenting their propensity to select among nutritionally distinct prey [29].

In three laboratory experiments we examined the potential impact of nutrition in driving the observed foraging patterns in the wolf spider *P. milvina*. Since *P. milvina* readily forage on small Diptera [27], [30], we used laboratory-reared fruit flies (*Drosophila melanogaster*) as prey and took advantage of well-established protocols that produce adult flies that differ nutritionally in ways that affect spider performance [1], [31], [32]. In Experiment 1, we tested the hypothesis that prey quality affects the proportion of prey killed and spider consumption across a wide range of prey densities. Because spiders grow bigger, survive longer and produce eggsacs more quickly on high quality prey [1], [31], [32], we predicted that *P. milvina* would kill and consume more of that prey type. In Experiment 2, tested the hypothesis that the dietary history of the spider would affect the proportion of prey captured and consumed. We predicted that spiders with a poor nutritional background would attempt to compensate for their recent diet [10], [14] and kill and consume more of the prey provided during the experiment. In Experiment 3, we tested the hypothesis that feeding history would influence prey selection by spiders when presented with a mixture of flies that differed in the nourishment they could provide. We predicted that, regardless of dietary experience, if the spiders could differentiate the prey types, they would select the high quality items. These experiments show that prey quality affects food intake but the dietary history of the spider shifts predatory intensity and alters prey preferences. Thus, nutrition has strong effects on predator-prey interactions and, as a result, it is likely to have dynamic impacts on the roles that carnivores play in different food webs and at different times.

## Materials and Methods

### Ethics statement

By using invertebrate species and caring for them using accepted ethical standards in the laboratory, our research conforms with legal requirements of the United States of America and guidelines established for the treatment of animals in research [33]. The species used for the experiments are not endangered or protected, and experimental animals were isolated from locations that are open to the public.

### Study system

Penultimate and adult female *P. milvina* were collected from the Ecology Research Center at Miami University, Butler County, Ohio (39°31′42″N, 84°43′48″W) between May and August 2006 for Experiments 1 and 2, and between May and August 2007 for Experiment 3. Once in the laboratory, *P. milvina* were housed individually in plastic containers (6 cm diameter, 4 cm deep) with a 1.5 cm layer of moist soil covering the bottom. Spiders were placed in an environmental chamber (25°C, 50–58% RH and 13∶11 L∶D cycle), fed two 0.32 cm crickets, and watered twice per week until they were randomly assigned to treatments. For all experiments, we used vestigial-winged fruit flies, *Drosophila melanogaster* (Diptera: Drosophilidae), as the prey species. Studies have shown that Diptera are an important component in the diet of wolf spiders [30], [34] and the nutritional content of Diptera can vary substantially in the field [35], [36]. In addition, there is a well-established approach to altering the nutritional content of *Drosophila* by adjusting the media in which they are cultured and the resulting differences directly affect the growth, performance, and behavior of *Pardosa* species [1], [31], [32], [37], [38].

### Prey manipulations and nutritional analyses

We generated two nutritional types of *Drosophila* for these experiments, which we refer to as low and high quality based on previous studies of their affects on *Pardosa* growth and survival [1], [31]. Our low quality flies were raised on standard commercial media (Instant *Drosophila* Media, Ward's™ Natural Science, Rochester, NY, USA) and our high quality prey were reared on the same media supplemented with 40% dog food by mass (Ol' Roy™ Dog Food, Wal-Mart, Bentonville, AR, USA). To determine if culture environment affected adult fly mass, five flies from seven different culture vials flies were dried to 60°C for 48 hrs and weighed to the neared 0.001 mg on a microbalance. We also quantified the carbon and nitrogen content of flies selected from 19 low quality cultures and from 20 high quality cultures. These flies were dried at 60°C for 48 hrs, packaged into 2–3 mg units (6–8 flies from a single culture bottle), and processed using a CHN analyzer (Perkin Elmer, Boston, MA, USA). From this, we obtained values of percent carbon(C), percent nitrogen (N) and the carbon to nitrogen ratio (C∶N).

In 2011, we conducted follow up analyses to estimate lipid and protein differences between flies reared in comparable circumstances. For these assays, the flies were reared in culture media prepared as described above except that Pedigree© Adult Complete Nutrition Dog Food was used for the high quality group (Mars Australia, North Ryde, Australia). Emerging adult flies were collected from 13 cultures and stored at −20°C prior to nutrient analyses. We used a subsample of 20 adult female flies that were haphazardly selected from containers to which we were blind of the experimental treatment. We measured lipid content gravimetrically by taking the difference in the dry weight of samples before and after two 24 hr soaks in chloroform. We then measured protein content using the Bradford Assay modified for use in 96 well microplates [39]. Protein was extracted from lean, ground samples using 0.1 M NaOH and heat (90°C for 30 minutes) after which samples were centrifuged and the supernatant was collected for analysis. We analyzed each sample in triplicate and all samples were run together on the same plate with a calibration curve created using IgG as a standard. We then calculated the percent lipid, percent protein and the ratio of lipid to protein.

### Protocols common to all experiments

All experiments were conducted in circular plastic arenas (20 cm diameter, 10 cm deep) with a 2 cm layer of moist potting soil mixture covering the bottom, which helped to maintain humidity, and a 3 cm layer of artificial straw (Textraw® Synthetic Straw, St. Simons Island, GA, USA), which provided habitat structure. In spiders, the size of the cephalothorax is fixed and only changes when they molt but the size of the abdomen varies with recent consumption. As such, any change in abdomen size can be used to quantify consumption over short periods of time [40]–[42]. Just prior to the experiment, we measured the carapace and abdomen width of all *P. milvina* using a digital micrometer attached to a stereomicroscope, accurate to ±0.01 mm, so that we could verify that spiders assigned to treatments did not differ in size or condition. At the commencement of each trial, we released the flies, allowed them to disperse for 15 min and then introduced a single spider. The container was closed, and placed in an environmental chamber (13 Light∶ 9 Dark, 25°C, 40–50% RH). After 24 hrs, we removed the spider, re-measured the abdomen width, counted the flies that were still alive as well as any partially consumed prey carcasses still in the arena. Any other missing flies were scored as killed by the spider. Spiders were never reused within or between experiments. The soil was discarded after use. The containers and artificial straw were cleaned with detergent, rinsed, wiped down with alcohol, and allowed to dry completely before reuse in subsequent trials.

### Experiments

Experiment 1 aimed to test the hypothesis that spiders shift their foraging intensity or consumption when presented with prey that differed in quality. To ensure the spiders were in similar condition, similar nutritional status, and had no recent experience with the flies we were using as prey in the experiment, we fed them two 0.32 cm crickets, twice per week for two weeks prior to testing. We quantified the predation and consumption by *P. milvina* presented with either low or high quality fruit flies at five densities (10, 20, 30, 40, 50; n = 10–15/treatment).

Experiment 2 was designed to test the hypothesis that recent dietary experience influences predation and consumption by *P. milvina*. We standardized spider experience and adjusted their nutritional status by providing them with either two low or two high quality flies twice per week for three weeks prior to testing. We quantified predation success and consumption by both groups of spiders at five densities of high quality flies (10, 20, 30, 40, 50; n = 10–15/treatment).

With Experiment 3, we tested the hypotheses that *P. milvina* could discriminate between prey that differed in nutritional composition and that recent feeding history affects their prey choice. We used two fruit fly mutants, red-eyed and white-eyed, so that we could distinguish between prey types. We conducted a preliminary choice test in order to establish that *P. milvina* did not prefer one mutant over the other. The spiders for these trials had been on the standard laboratory diet of two 0.32 cm crickets, twice per week, for several weeks and had no prior experience with either fly type. We presented one group of spiders with 20 white-eyed low quality flies mixed with 20 red-eyed high quality flies and a second group 20 white-eyed high quality flies mixed with 20 red-eyed low quality flies (n = 11/group). Spiders were allowed to forage on the 40 flies for a 24-hr period and then they were removed. The remaining flies were counted and categorized by eye color. Following these trials, we selected different set of spiders from laboratory cultures and placed them on a diet of either two low (n = 20) or two high (n = 21) quality flies twice per week for three weeks. We then allowed these animals to forage in a mixed prey environment containing 20 white-eyed low quality flies mixed with 20 high quality red-eyed flies for 24 hrs and counted and categorized the remaining flies as alive or dead but not eaten.

### Data analysis

#### Prey manipulations and nutritional analysis

We compared the dry mass, percent C, percent N and C∶ N ratio of the two types of flies reared in identical cultures to those used in experiments using an ANOVA with culture bottle as a blocking factor. For the follow-up analysis where we quantified percent lipid, percent protein and the lipid to protein ratio of a different set of flies, we also used one-way ANOVA to compare those reared in regular and dog food amended media.

#### Spider size and condition

In order to verify that the animals assigned to different treatments for the same experiment did not differ in size, we compared the carapace width, a measure that cannot change except when an animal molts, among treatments within each experiment using one-way ANOVAs. We established that spiders in different treatment groups were in similar body condition at the outset of each experiment by comparing abdomen width among treatments in ANCOVAs with carapace width as a covariate to account for spider size.

#### Experiments 1 & 2: The functional response

We examined the relationship between the number of prey provided to the spiders and the number captured using a well-established two-step approach to analyzing the functional response [43] (see also [25], [44], [45]). Specifically, the function that linked the total number of prey provided (*N_0_* = initial number of flies) and the proportion of prey killed (*N_e_/N_0_*; where *N_e_* = number killed, and *N_0_* = initial number of flies) was characterized using logistic regression (PROC LOGISTIC in SAS 9.2, SAS Institute, Inc., Cary NC, USA). We included a quadratic term (*N_0_^2^*) to account for nonlinearity, however it is the linear component of this function that determines the type of functional response. Specifically, a type I functional response would be identified if the proportion of prey killed varied as a linear function of prey density (*N_0_*), and the slope of the regression was not significantly different from zero, a type II functional response would be identified if the slope was significantly less than zero, and a type III would have a slope significantly greater than zero. Separate models were fit for each experiment and indicator variables for prey type (Experiment 1) or spider diet (Experiment 2) allowed us to evaluate treatment differences in the function. We followed that analysis with an iterative approach that takes into account the fact that prey were depleted during the course of the experiments to estimate the attack constants (*a*) and handling times (*T_h_*) [43], [46]. Specifically, we used the integrated form of Holling's disc original equation [28], which is referred to as the random predator equation: *N_e_* = *N_0_{1−exp[a(T_h_ N_e_−T)]}*; where *N_e_* is the number of flies killed, *N_0_* is the initial fly density, *a* is the attack constant (instantaneous rate of discovery), *T_h_* is handling time (time for pursuing, attacking, catching and eating) and *T* is total time. We solved for *a* and *T_h_* using nonlinear least squares and with Newton's method for parameter estimation (PROC NLIN, SAS 9.2, SAS Institute, Inc., Cary, NC USA); an approach that has been shown to provide more accurate estimates of these functional response parameters than linear models [43], [46]. Indicator variables were added to the implicit function so that we could compare parameters between the treatments within each experiment using t-tests and confidence intervals.

#### Experiments 1 & 2: Prey consumption

To assess the effects of our treatments on prey consumption, we calculated the change in abdomen width that occurred during the 24 h duration of feeding in each experiment (after – before) (as in [41]) and compared these values across treatments in two-way ANOVAs. We also used two-way ANOVAs to look for treatment differences in the number of partially consumed prey found at the end of each experiment.

#### Experiment 3: Prey choice

We used the Manly-Chesson selectivity metric, α, to assess prey choice [47], [48]. Specifically, the equation to estimate α for low quality prey is; *α = ln((n_1_−r_1_)/n_1_)/[ln((n_1_−r_1_)/n_1_)+ln ((n_2_−r_2_)/n_2_)]* where *n_1_* and *n_2_* equal the initial number of low or high quality flies, *r_1_* and *r_2_* is the number of low or high quality flies consumed. To compare selectivity of the two fly types between the two prior diet treatments, we calculated α's and 95% confidence intervals (CIs) for prior diet treatments for each prey type (low and high quality prey). We concluded that the spiders preferred one prey type over the other when the CI was above the α = 0.5 point, that they avoided a prey type when the CI was below the α = 0.5 point, and that there was no preference when the CI overlaps with α = 0.5. As a further test, the null hypothesis of no preference among the two prey types within a treatment (i.e. α high quality = α low quality = 0.5) was compared against the alternative of α high quality ≠α low quality using Hotelling's *T^2^* (i.e. multivariate t-tests) because of the inherent dependence in the α vector (i.e. Σα_i_ = 1; [48]). We used one-way ANOVAs to compare the number of high vs. low quality prey that were killed but only partially consumed for each diet treatment. All analyses were conducted in SAS 9.2 and JMP 8.0 (SAS Institute, Inc., Cary, NC, USA).

## Results

### Nutritional analysis

There was no difference in the dry mass of fruit flies reared on the different media (Table 1). While the percent C was similar for flies in the two culture types, percent N was significantly higher and the C∶N ratio was significantly lower for high quality than for low quality flies (Table 1). The follow-up analyses of fly nutritional content revealed that high quality flies had higher protein levels but less lipid than what was measured for low quality flies (Table 1). As a result, the lipid to protein ratio was also significantly lower for high quality flies (Table 1).

**Table 1:** Average (± SE) nutrient content and results of ANOVAs comparing of the low quality fruit flies (reared on the standard *Drosophila* media) and high quality flies (reared on media supplemented with dog food media).

Variable	Low quality	High quality	*F*	*P*
Mass	0.334±0.012	0.310±0.016	2.16'	0.15
Percent C	51.81±0.21	51.62±0.14	0.53*	0.47
Percent N	8.92±0.14	9.32±0.14	4.34*	0.04
C∶N	6.79±0.11	6.48±0.10	4.52*	0.04
Lipid	23.3±0.43	20.3±1.16	5.96∧	0.02
Protein	54.0±0.94	58.0±1.46	5.31∧	0.03
Lipid∶Protein	0.43±0.01	0.36±0.03	6.94∧	0.01

Symbols indicate:

' *F_1,56_*;

* *F_1,36_* and

∧ *F_1,24_*.

### Spider size and condition

The spiders that we assigned to treatments did not differ in carapace width, our measure of spider size, in any of the experiments (Table S1). Likewise, the condition of animals in the different treatment groups, as measured by the abdomen width with a control for total size, was the same at the commencement of each experiment (Table S1). Hence, a significant change in abdomen size related to treatment can be attributed to differential consumption under the experimental conditions.

### The functional response

**Figure 1:**
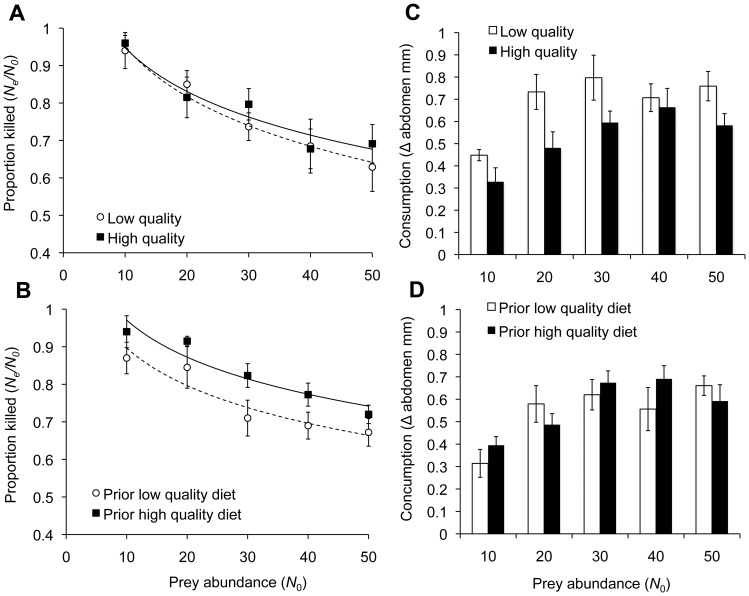
Effects of prey type and predator prior diet on the proportion killed and amount consumed. **A:** The proportion of prey killed was not affected by prey type (i.e. whether they were tested with low or high quality flies) (Experiment 1). The values indicated by symbols represent the average proportion (±1SE) with open circles and dotted lines corresponding to results with low quality prey and solid circles and lines representing results with high quality prey. **B:** The proportion of prey killed was influenced by whether the spider had been maintained on a low or high quality diet prior to testing (Experiment 2). The values indicated by symbols represent the average proportion (±1SE) with open circles and dotted lines corresponding to results for spiders on low quality diets prior to testing and solid circles and lines representing results for spiders on prior high quality diets. **C:** Consumption, as represented by average (±1SE) change in abdomen width of the spider, was affected by prey type (Experiment 1). Open bars indicate consumption of low quality prey and solid bars indicate consumption of high quality prey. **D:** Consumption, as represented by average (±1SE) change in abdomen width of the spider, was not influenced by the prior diet of the spider (Experiment 2). Open bars indicate consumption of spiders on prior low quality diet and solid bars indicate consumption of spiders on prior diet of high quality prey.

**Figure 2:**
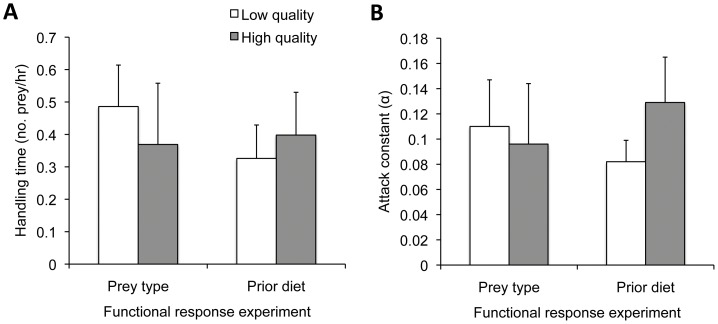
Estimates of functional response parameters: handling times (**A**) and attack constants (**B**). In experiment 1, there were no differences in **A:** handling time (df = 1,96, t = 0.51, P = 0.61) or **B:** attack constant (df = 1, 96, t = 0.03, P = 0.82) between spiders foraging on low or high quality prey. In experiment 2, there were no differences in **A:** handling time (df = 1,98, t = 0.43, P = 0.67) or **B:** attack constants (df = 1, 98, t = 01.18, P = 0.24) for spiders with contrasting dietary histories. Bars indicate averages with 95% CI. Open bars are used for low quality prey type or diet and shaded bars are used for high quality prey type or prior diet.

In all cases, there was a strong decline in the proportion of prey consumed with increasing prey density regardless of the quality of prey used in testing (Experiment 1) or the content of the spider's diet in the weeks prior to testing (Experiment 2) (Table 2, Figs. 1A, 1B). Thus, *Pardosa milvina* consistently displayed a Type II functional response in our experimental situation. Likewise, there were no effects of prey quality (Experiment 1) or prior diet (Experiment 2) on the calculated attack constants (*a*) or handling times (*T_h_*) (Fig. 2).

**Table 2:** Results of logistic regression used to determine the type of functional response for each experiment and to test for differences between either prey type or spider prior diet.

	Estimate	*SE*	*χ^2^*	*P*
Prey type				
Intercept	3.368	0.402	81.88	<0.0001
*N_0_*	−0.119	0.024	25.08	<0.0001
*N_0_^2^*	0.001	0.000	12.83	0.0003
Prey treatment	0.076	0.043	3.16	0.0756
Prior Diet				
Intercept	2.805	0.315	79.28	<0.0001
*N_0_*	−0.072	0.019	13.89	0.0002
*N_0_^2^*	0.001	0.000	6.00	0.0143
Prey treatment	0.201	0.039	26.79	<0.0001

Estimates of coefficients and standard error (SE) are presented for initial prey abundance, *N_o_*, *N_o_^2^*, and prey treatments.

### Experiment 1: Prey quality

*Pardosa milvina* killed similar amounts of high and low quality prey over the range of prey densities tested (Table 2, Fig. 1A). However the lines that characterized the functional responses appear to be diverging at high prey densities (Fig. 1A) and the p-value is marginal at 0.076, so animals might kill a greater proportion at very high prey densities. Interestingly, there was a significant effect of prey nutrient content on spider consumption, as measured by the amount the abdomen changed in size during the course of the experiment (Table 3, Fig. 1C). Spiders foraging on low quality flies increased ca. 30–50% more than those provided high quality flies in the lower three prey densities (10, 20 & 30 flies). However, the amount that abdomen width changed was similar for animals foraging on both prey types at the highest prey densities (40 & 50 flies), suggesting that consumption reached some threshold (Fig. 1C). There was no difference in the number of low vs. high quality prey that were left partially consumed, however there were more carcasses left behind at the higher initial prey densities (Table S2).

**Table 3:** Results from ANOVA used to determine the effect of prey type or spider prior diet on prey consumption, as measured by the change in abdomen width of *Pardosa milvina* during the course of the experiment.

Source	*df*	SS	*F*	*P*
Prey type				
Prey treatment	1	0.23	15.21	0.0002
Initial prey abundance (*N_0_*)	4	0.61	10.16	<0.0001
Interaction	4	0.03	0.54	0.70
Residual	96	1.34		
Prior diet				
Prey treatment	1	0.02	0.64	0.43
Initial prey abundance (*N_0_*)	4	0.74	7.22	<0.0001
Interaction	4	0.12	1.19	0.32
Residual	98	2.99		

### Experiment 2: Predator diet

When *P. milvina* was provided high quality prey in the weeks prior to testing, they killed a significantly greater proportion of prey across all densities than those on a diet of low quality flies (Table 2, Fig. 1B). Consumption, as measured by the change in abdomen width over the course of the experiment, increased with the number of prey provided and leveled off at the higher prey densities but was not significantly influenced by the treatments (Table 3; Fig. 1D). Spiders with different nutritional histories left similar numbers of partially consumed prey but there were more carcasses when initial prey densities were at the highest levels (Table S2).

### Experiment 3: Prey choice

**Figure 3:**
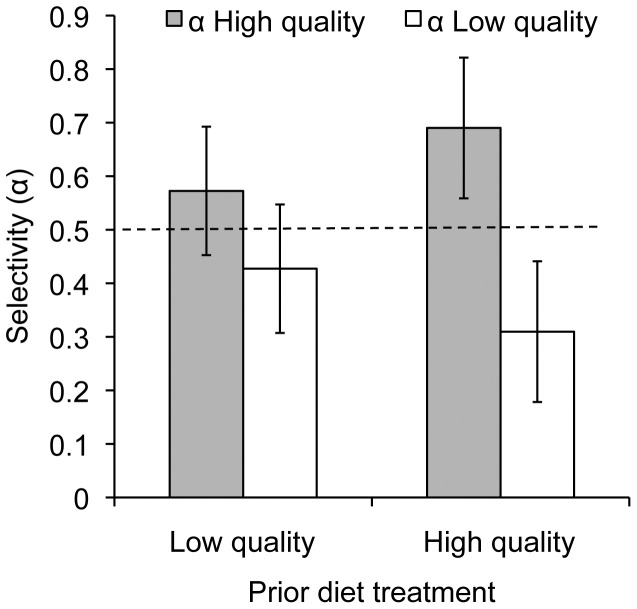
Prey type selected by spiders that were provided either a low or high quality diet. In experiment 3, the average preference scores (α) (and 95% confidence interval) for spiders that had been on a low quality diet were not significantly different from α = 0.50, which represents random prey selection (dashed horizontal line), whereas spiders on a high quality diet prior to testing showed negative selection for low quality flies (95% CI lies below the line) and positive selection for high quality flies (95% CI is above the line).

Initial choice trials revealed that spiders did not differentiate between the fly phenotypes (i.e. red-eyed vs. white-eyed) (Hotelling's *T^2^* = −0.42, *df* = 21, *P* = 0.67), which allowed us to use these mutants to discriminate flies that differed in quality in subsequent tests. Spiders previously maintained on a low quality diet had selectivity scores for both low and high quality flies that overlapped the no preference line indicating that they did not actively choose prey based on content (Hotelling's *T^2^* = 1.41, *df* = 14, *P*<0.27, Fig. 3). However, the spiders that had been maintained on high quality flies exhibited significant positive selection for high quality flies while selecting against low quality flies (Hotelling's *T^2^* = 6.90, *df* = 13, P<0.021, Fig. 3). There were no differences between the number of high vs. low quality prey that were found dead but not fully consumed for either treatment group (Table S2).

## Discussion

Here we show that the dietary history of an arthropod predator had strong effects on foraging behavior whereas differences in the nutritional content of the prey seemed to primarily affect intake. Specifically, spiders with similar nutritional histories killed similar numbers of high and low quality prey (Fig. 1A) but ingested more of the low quality prey (Fig. 1C). While spiders that had been foraging on high quality prey in the weeks before the experiment killed significantly more flies across all prey densities than those foraging on low quality prey (Fig. 1B), we documented no differential consumption (Fig. 1D). Surprisingly, wolf spiders were also able to discriminate between prey based solely on their nutrient content (Fig. 3), which suggests that prey choice in field situations occurs on a finer scale than is typically considered. Indeed, these results may explain why predation by generalist predators scales with prey abundance in some situations and not in others [27], [49], [50]. It is becoming increasingly evident that nutrition has sizeable consequences on trophic interactions and that incorporating more detailed dietary information into models of food webs will provide insight into the structure and function of communities and ecosystems [4], [5].

Differences in the protein and lipid content of currently available prey as well as the prey provided to the spider in the recent past affected foraging behavior of the wolf spider, *P. milvina*. We used an approach to manipulating prey content derived from a number of previous studies where, in every case, performance (e.g., growth and reproduction) of spiders fed flies cultured in media augmented with dog food was significantly higher than performance of spiders fed flies reared in standard media [1], [31], [37]. Our high quality flies differed primarily in nitrogen content which typically translates directly to crude protein content [51], however other studies demonstrated that fruit flies reared in dog food amended media were better prey for spiders than those in which protein was manipulated in isolation [31]. While it would be nice to isolate behavioral responses to specific compositional differences, in nature, prey will undoubtedly present highly variable packages of macro- and micronutrients that interact with one another and the feeding history of the consumer. Here we chose to maximize the nutritional differences of the prey while minimizing any other differences such as prey species or size.

Prey quality did not affect the number of prey that *P. milvina* killed, but it did influence the amount consumed by the spiders, as evidenced by the change in abdomen size (Figs. 1A, C). The protein leverage hypothesis predicts that consumers will continue to feed until they reach some target protein intake level [6], [52] and recent studies show that spider predators can adjust intake to maintain specific amounts of protein and lipid [10], [14], [53]. In Experiment 1, we standardized the diet of the test spiders using crickets in order to focus on nutritional differences and eliminate any affect of experience with the prey species. However the crickets provided to these spiders in the weeks prior to testing contain relatively high levels of both protein and lipid, which made them similar in protein (ca. 58%) and higher in lipid (ca. 27%) than our high quality flies [54]. Thus, the higher consumption of the low quality prey with less protein than the high quality flies may be due to the spider's drive to increase protein consumption in order to balance their diet. Even if the animals were attempting to match their recent nutrient intake they would have to extract more material to get similar amounts of protein from the low quality flies. Although differential digestibility of low and high quality flies could bias measurements of abdomen width as a proxy for the biomass of flies ingested, a study of a congeneric spider, *P. prativaga*, showed that, once spiders ingest prey the rate at which body mass is lost over a short term period (i.e., <5 days) is independent of the nutrient content of the prey [53]. Thus, we believe that the changes observed over the 24 hr of our experiments reflect real differences in intake that indicate the spider's response to the nutritional content of their prey.

We had anticipated that the animals maintained on low quality diet would have higher kill rates when confronted with an abundance of high quality prey items, however the opposite occurred and feeding experience with low quality prey reduced the proportion of prey taken across the range of densities we tested (Fig. 1B). Additionally there is no evidence that the animals on the low quality diet compensated through differential ingestion to the extent we could quantify it through changes in abdomen size (Fig. 1D). In previous studies, wolf spiders fed high quality fruit flies were more likely to take prey [31], tended to be more cannibalistic [31] and attacked potential mates more frequently [32]. Thus, it seems likely that nutritional differences of these fruit fly diets affects *P. milvina* foraging success either by altering the inherent aggressive tendencies of individuals or impacting their physiological state in a way that influences stamina or motivation.

It is interesting to note that, despite differences in intake in one of our functional response experiments (Experiment 1) and predatory intensity in the other (Experiment 2), there were no significant treatment effects on the estimated handling times or the attack constants in either experiment (Fig. 2). Intuitively, we expected that the time the predators took to subdue and feed on a given prey item would be affected by the availability of additional possible prey items and that the observed differences in kill rates between treatments would be reflected in the attack constants or handling times. However, the small fruit flies seemed to be easy prey for *P. milvina* and we regularly observed spiders capturing and handling several of them at the same time. Likewise, the numbers of partially consumed prey carcasses suggest that spiders killed more prey than they could consume during the 24 h of our trials, which may mean that they would have returned to eat more if they had been left for longer periods of time. Indeed superfluous killing at high prey densities is common in many species of spiders and is presumed to have resulted because they are adapted to severe food limitation [55]–[57]. Taken together, all of these factors likely acted in concert to reduce any possible differences in the attack rates and handling times generated from our functional response data in Experiments 1 and 2.

Another recent study examined the foraging response of larger wolf spider species, *Pardosa amentata*, to fruit fly prey differing in protein and lipid content [23]. While they present their data as a study of the functional response, the approach and analysis are rather unconventional. Nevertheless, there were a few days over the course of their experiments where *P. amentata* captured different numbers of high protein and high lipid flies and, to the extent that they occurred, the preferences differed between juveniles and adults. While the many differences between their study and ours preclude any direct comparison, the results of both underscore that consideration of nutritional factors when attempting to understand the role of predators in food webs is complex but critical to our understanding of species interactions.

The ability of *P. milvina* to select among a mixture of high and low quality fruit flies was one of our most intriguing findings (Fig. 3). Foraging wolf spiders are sensitive to the movement of prey items [58] and, if the activity of prey was differentially affected by their culture environment, then that could account for differential captures. However, only spiders on the high quality diet selected among prey types and no parsimonious explanation would allow us to postulate that predator diet would shift their relative reliance on, or sensitivity to, prey activity while foraging. On the other hand, *P. milvina* is highly attuned to their chemical environment, which is important to their ability to detect prey [59], predators [60], [61] and potential mates [62], [63]. This sensitivity suggests that *P. milvina* is likely to use chemical information to discriminate and select among the prey types. Specifically, animals with experience on the good diet responded to the chemical signature of the familiar prey but those on a poor diet foraged indiscriminately on the prey known to be of low quality and those of unknown nutritional value (Fig. 3).

Our study demonstrates the need for a more explicit integration of prey nutritional composition and predator dietary history into the metrics of foraging, such as the functional response [29]. We show that, even if prey size and prey species identity are controlled, recent feeding history influences the functional response in non-intuitive ways. For example, we might have assumed that a poorly fed predator would have had the largest impact on prey populations but we discovered that predators fed on high quality diets removed a greater proportion of the prey population (Fig. 1A). In addition, while historically, the theory that includes nutritional interactions in the functional response suggests there should be a preference for rare food sources [64], that is not what we observed here. More recent nutritionally explicit approaches reveal compromise rules for intake depending on how close the prey are to some optimal composition [5], [6], [15]. Our results contribute to a lively research area, which posits that the role of a given predator in the food web is dependent on the nutritional landscape mediated through changes in behavior and physiology of both the predator and its potential prey.

## Supporting Information

Table S1 Results from analyses to determine the equivalence of spider size and condition across treatments.
(DOCX)

Table S2 Summary statistics and analyses of the number of partially consumed prey left behind in experiments.
(DOCX)
