# Supplementary material for: The Nutritional Content of Prey Affects the Foraging of a Generalist Arthropod Predator
Source: PLoS One. 2012 Nov 8;7(11):e49223. doi: 10.1371/journal.pone.0049223 (PMC3493534; doi:10.1371/journal.pone.0049223)
Supplement: Table S1 — Results from analyses to determine the equivalence of spider size and condition across treatments. (DOCX) [file pone.0049223.s001.docx]

**Table S1: Results from analyses to determine the equivalence of spider size and condition across treatments.**

Initial measurements of spiders, *Pardosa milvina*, used to confirm that there were no differences in the size (ANOVA; carapace width, mm), and body condition (ANCOVA; abdomen width, mm, relative to carapace width, mm; multiple regression model) of individuals assigned to the experimental treatments.

| Source | Mean ± 1SE | *df* | *F* | *P* |
| --- | --- | --- | --- | --- |
| Experiment 1 |  |  |  |  |
| Spider size |  | 1, 96 | 0.25 | 0.62 |
| Low quality | 2.13 ± 0.03 |  |  |  |
| High quality | 2.14 ± 0.03 |  |  |  |
| Body condition |  |  |  |  |
| Carapace (covariate) |  | 1, 96 | 27.61 | <0.0001 |
| Treatment |  | 1, 96 | 0.29 | 0.59 |
| Low quality | 1.89 ± 0.04 |  |  |  |
| High quality | 1.86 ± 0.04 |  |  |  |
| Experiment 2 |  |  |  |  |
| Spider size |  | 1, 98 | 1.64 | 0.20 |
| Low quality | 2.16 ± 0.02 |  |  |  |
| High quality | 2.13 ± 0.02 |  |  |  |
| Body condition |  |  |  |  |
| Carapace (covariate) |  | 1, 98 | 13.56 | 0.0003 |
| Treatment |  | 1, 98 | 0.70 | 0.41 |
| Low quality | 2.03 ± 0.03 |  |  |  |
| High quality | 1.99 ± 0.03 |  |  |  |
| Experiment 3 |  |  |  |  |
| Spider size |  | 1, 41 | 1.60 | 0.22 |
| Low quality | 2.09 ± 0.04 |  |  |  |
| High quality | 2.01 ± 0.01 |  |  |  |
| Body condition |  |  |  |  |
| Carapace (covariate) |  | 1, 24 | 5.39 | 0.03 |
| Treatment |  | 1, 24 | 1.14 | 0.30 |
| Low quality | 1.98 ± 0.07 |  |  |  |
| High quality | 2.00 ± 0.08 |  |  |  |
