# Supplementary material for: The Nutritional Content of Prey Affects the Foraging of a Generalist Arthropod Predator
Source: PLoS One. 2012 Nov 8;7(11):e49223. doi: 10.1371/journal.pone.0049223 (PMC3493534; doi:10.1371/journal.pone.0049223)
Supplement: Table S2 — Summary statistics and analyses of the number of partially consumed prey left behind in experiments. (DOCX) [file pone.0049223.s002.docx]

**Table S2: Summary statistics and analyses of the number of partially consumed prey left behind in experiments.**

Experiment 1

No difference in partial consumption of prey between the two prey quality treatments (*F_1, 96_* = 0.29, *P* = 0.59). Partial consumption did differ between fly abundance treatments (*F_4, 96_* = 15.87, *P* < 0.0001) in a similar way across the fly quality treatments as indicated by no significant interaction (*F_4, 96_* = 0.93, *P* = 0.45).

| Quality treatment | Abundance treatment | Mean partial consumed ±1SE |
| --- | --- | --- |
| Low quality | 10 | 0.33 ±0.33 |
|  | 20 | 0.83 ±0.54 |
|  | 30 | 5.00 ±1.65 |
|  | 40 | 4.00 ±1.37 |
|  | 50 | 7.50 ±3.93 |
| High Quality | 10 | 0.67 ±0.49 |
|  | 20 | 3.20 ±1.39 |
|  | 30 | 1.50 ±0.87 |
|  | 40 | 4.50 ±2.02 |
|  | 50 | 3.50 ±1.50 |

Experiment 2

A prior diet of prey differing in quality did not have a significant effect on partial prey consumption (*F_1, 98_* = 0.07, *P* = 0.80). Partial consumption did differ between fly abundance treatments (*F_4, 98_* = 19.09, *P* < 0.0001) in a similar way across the fly quality treatments as indicated by no significant interaction (*F_4, 98_* = 0.44, *P* = 0.78).

| Prior quality treatment | Abundance treatment | Mean partial consumed ±1SE |
| --- | --- | --- |
| Low quality | 10 | 0.80 ±0.35 |
|  | 20 | 1.25 ±0.35 |
|  | 30 | 2.67 ±0.69 |
|  | 40 | 7.31 ±2.05 |
|  | 50 | 10.08 ±1.85 |
| High quality | 10 | 1.00 ±0.36 |
|  | 20 | 2.00 ±0.52 |
|  | 30 | 5.08 ±0.99 |
|  | 40 | 7.08 ±1.16 |
|  | 50 | 13.67 ±2.20 |

Experiment 3

No difference in the partial consumption of low quality (*F_1, 23_* = 0.03, *P* = 0.86), or high quality (*F_1, 23_* = 0.21, *P* = 0.65) as related to prior diet treatment before the choice test.

| Prior quality treatment | Mean low quality partial consumed ±1SE | Mean high quality partial consumed ±1SE |
| --- | --- | --- |
| Low quality | 2.77 ±0.59 | 4.77 ±1.21 |
| High Quality | 2.67 ±0.63 | 3.75 ±1.02 |
